# Author Correction: Damaged brain accelerates bone healing by releasing small extracellular vesicles that target osteoprogenitors

**DOI:** 10.1038/s41467-025-61622-3

**Published:** 2025-07-03

**Authors:** Wei Xia, Jing Xie, Zhiqing Cai, Xuhua Liu, Jing Wen, Zhong-Kai Cui, Run Zhao, Xiaomei Zhou, Jiahui Chen, Xinru Mao, Zhengtao Gu, Zhimin Zou, Zhipeng Zou, Yue Zhang, Ming Zhao, Maegele Mac, Qiancheng Song, Xiaochun Bai

**Affiliations:** 1 Guangdong Provincial Key Laboratory of Bone and Joint Degeneration Diseases, Department of Cell Biology, School of Basic Medical Sciences, Southern Medical University, Guangzhou, 510515 China; 2 State Key Laboratory of Organ Failure Research, Academy of Orthopedics, Guangdong Province, The Third Affiliated Hospital of Southern Medical University, Guangzhou, Guangdong 510630 China; 3 Department of Radiology, Nanfang Hospital, Southern Medical University, Guangzhou, Guangdong 510515 China; 4 Department of Clinical laboratory, Nanfang Hospital, Southern Medical University, Guangzhou, Guangdong 510515 China; 5 Department of Pathophysiology, Guangdong Provincial Key Laboratory of Shock and Microcirculation Research, Southern Medical University, Guangzhou, Guangdong 510515 China; 6 Institute for Research in Operative Medicine, Private University of Witten-Herdecke, Cologne Merheim Medical Center, Ostmerheimerstr 200, D-51109 Cologne, Germany

Correction to: *Nature Communications* 10.1038/s41467-021-26302-y, published online 15 October 2021

In the version of the article initially published, in Fig. 4f, the “PTD (FOXO4)” image was misused from the “PTD + TBI (14d)” group in Fig. 1f. In Fig. 7h, the “Blank-14d image was misused from the group “PTD + TBI” in Fig. 2i, while the image for the group “Gel-14d” in Fig. 7h was misused from “PTD + TBI + GW4869” in Fig. 2i. The figures have been corrected in the HTML and PDF versions of the article.

