# Supplementary material for: Damaged brain accelerates bone healing by releasing small extracellular vesicles that target osteoprogenitors
Source: Nat Commun. 2021 Oct 15;12:6043. doi: 10.1038/s41467-021-26302-y (PMC8519911; doi:10.1038/s41467-021-26302-y)
Supplement: Supplementary file 1 — Supplementary Information [file 41467_2021_26302_MOESM1_ESM.pdf]

Supplementary Information

Damaged Brain Accelerates Bone Healing by Releasing Small Extracellular Vesicles that Target Osteoprogenitors

Wei Xia[1,7], Jing Xie[1,7], Zhi-Qing Cai[1,7], Xu-Hua Liu[2], Jing Wen[3], Zhong-Kai Cui[1], Run Zhao[1], Xiao-mei Zhou[1], Jia-Hui Chen[2], Xin-Ru Mao[4], Zheng-Tao Gu[5], Zhi-Min Zou[5], Zhi-Peng Zou[1], Yue Zhang[1], Ming Zhao[5], Maegele Mac[6], Qian-Cheng Song[1]★, Xiao-Chun Bai[1]★.

[1] Guangdong Provincial Key Laboratory of Bone and Joint Degeneration Diseases, Department of Cell Biology, School of Basic Medical Sciences, Southern Medical University, Guangzhou 510515, China

[2] State Key Laboratory of Organ Failure Research, Academy of Orthopedics, Guangdong Province, The Third Affiliated Hospital of Southern Medical University, Guangzhou, Guangdong 510630, China

[3] Department of Radiology, Nanfang Hospital, Southern Medical University, Guangzhou, Guangdong 510515, China

[4] Department of Clinical laboratory, Nanfang Hospital, Southern Medical University, Guangzhou, Guangdong 510515, China

[5] Department of Pathophysiology, Guangdong Provincial Key Laboratory of Shock and Microcirculation Research, Southern Medical University, Guangzhou, Guangdong 510515, China

[6] Institute for Research in Operative Medicine, Private University of Witten-Herdecke, Cologne Merheim Medical Center, Ostmerheimerstr 200, D-51109 Cologne, Germany

[7] These authors contributed equally: Wei Xia, Jing Xie, Zhi-Qing Cai.

★Correspondence: songqc@smu.edu.cn (Q.-C.S.), baixc15@smu.edu.cn (X.-C.B.)

SUPPLEMENTAL FIGURES

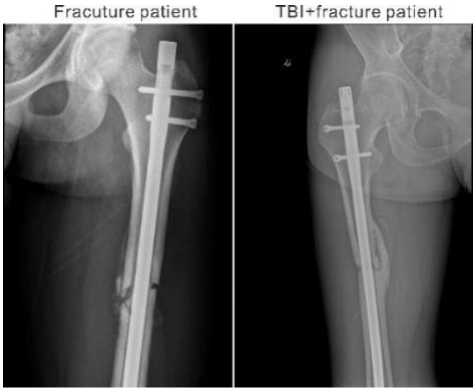

Supplementary Figure 1. The fracture recovery was much faster in patients with fracture and TBI (traumatic brain injury) than in patients with fracture only, which is related to Figure 1.

Representative X-ray images of fractures and concomitant TBI patients and fracture only patients.

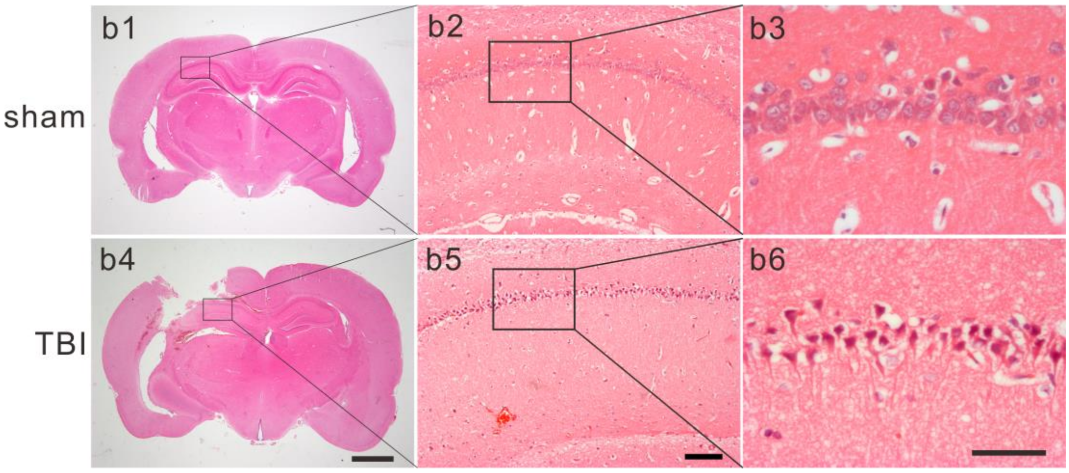

Supplementary Figure 2. Lateral fluid-percussion to establish the rat TBI model, which is related to Figure 1.

Representative hematoxylin and eosin staining of a gross (whole brain) and hippocampus (CA1) of sham and TBI rats. The b1 and b4 scale bars = 2,000 μm; b2 and b5 scale bars = 100 μm. The b3 and b6 scale bars = 50 μm. The representative data from three independent experiments are shown.

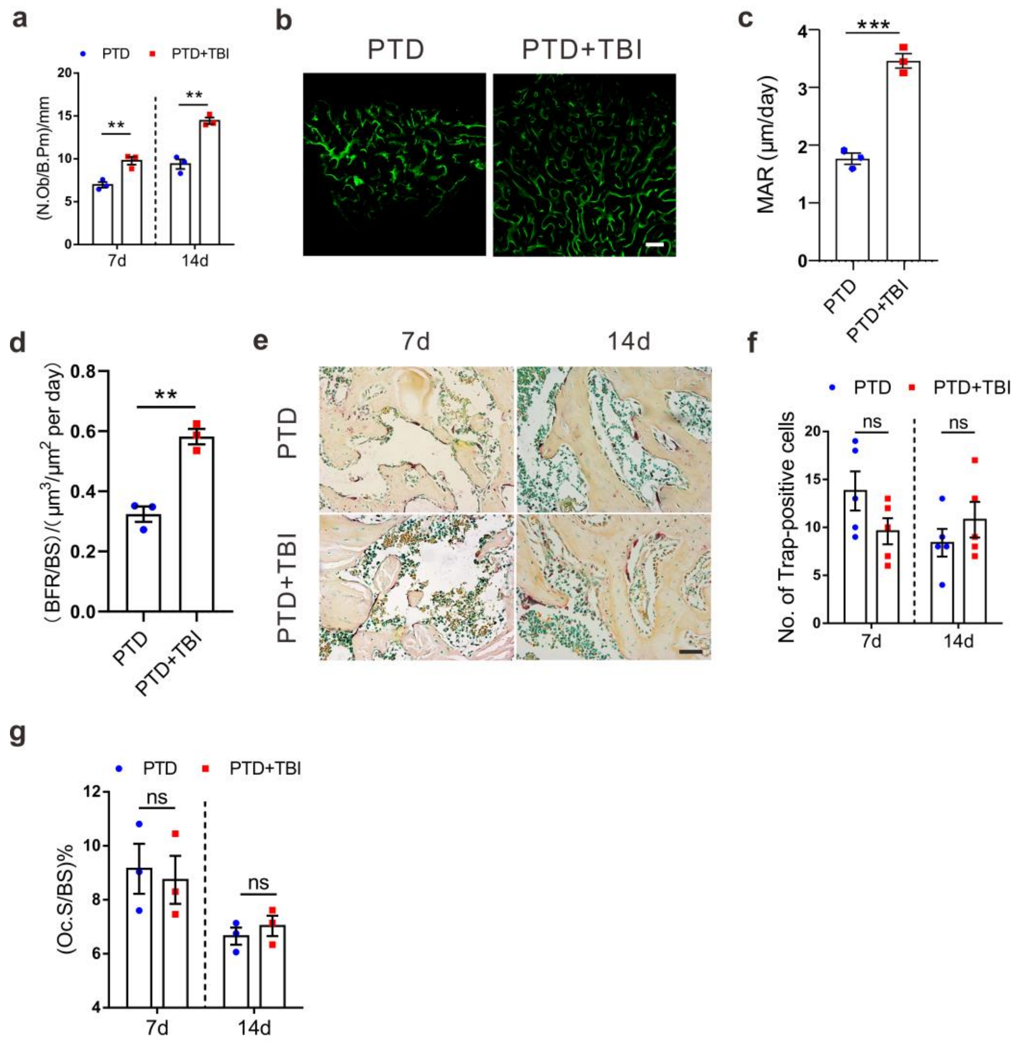

Supplementary Figure 3. There was no significant difference in the number of osteoclasts between the PTD (proximal tibia defect) group and PTD + TBI (PTD combined-TBI) group, which is related to Figure 1g.

The numbers of osteoblasts (**a**) per bone surface area were assessed (n = 3; Student's two-sided unpaired *t*-test). $P_{(7d\ PTD+TBI\ vs.\ PTD)}$=0.0074, $P_{(14d\ PTD+TBI\ vs.\ PTD)}$=0.0017. (**b**) Calcein were injected intraperitoneally to mark the new bone 1 and 3 weeks after the surgery. The defected tibia were harvested at week 4, and undecalcified sections were acquired using a Leica diamond saw. The calcein-labeled bone was observed by confocal microscopy (n = 3). Scale bar, 50 μm. The mineral apposition rate (MAR) (calculated in μm/day) (**c**) and their

bone-formation rate (BFR = MAR×minerization surface/bone surface) (**d**) are illustrated (n = 3; Student's two-sided unpaired *t*-test). $P_{(\text{MAR PTD+TBI vs. PTD})}$=0.0004, $P_{(\text{BFR/BS PTD+TBI vs. PTD})}$=0.0021. Representative TRAP staining (**e**), quantitation of TRAP-positive cells (**f**) and osteoclast surface per bone surface (**g**) in the osteoclasts around the defect space in the callus of PTD and PTD + TBI groups at 7 and 14 days after surgery (n = 5, Student's two-sided unpaired *t*-test). $P_{(\text{TRAP-positive cells 7d PTD+TBI vs. PTD})}$=0.1248, $P_{(\text{TRAP-positive cells 14d PTD+TBI vs. PTD})}$=0.3361, $P_{(\text{Oc.S/BS 7d PTD+TBI vs. PTD})}$=0.7662, $P_{(\text{Oc.S/BS 14d PTD+TBI vs. PTD})}$=0.4796. Scale bars: 50 µm. The quantitation result were plotted as dot plots, showing the mean ± SEM of three independent experiments; ns = non-significant.

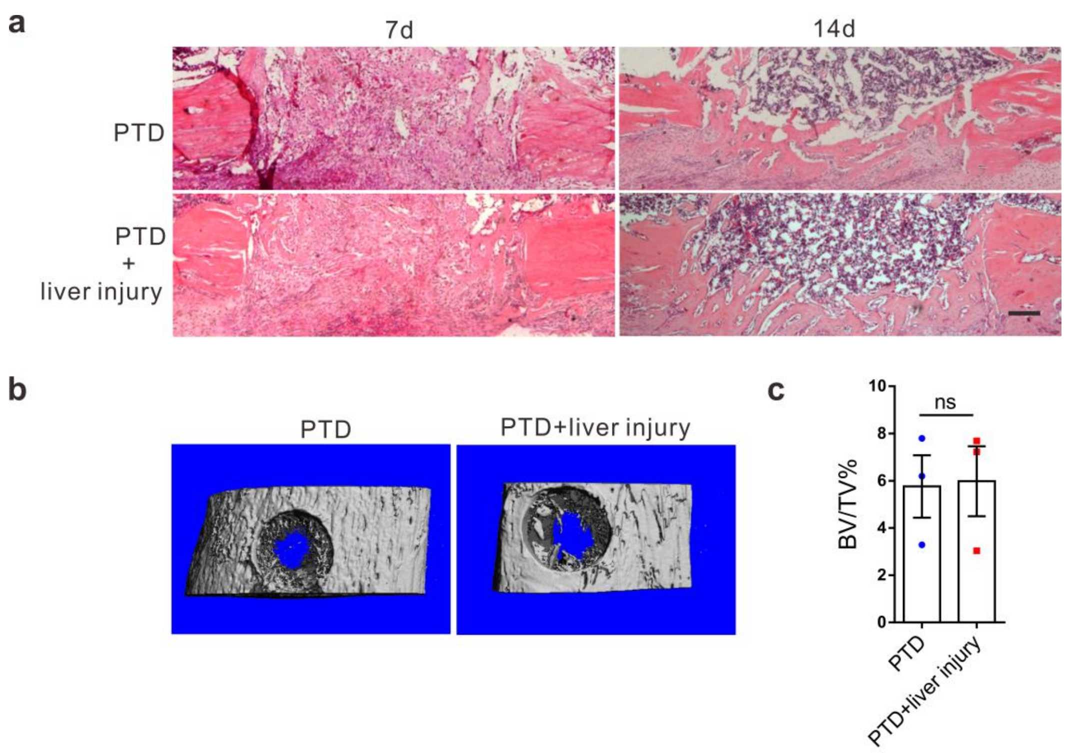

Supplementary Figure 4. Liver injury could not promote bone healing in a PTD combined with the liver injury model of rats, which is related to Figure 1.

(**a**) Representative hematoxylin and eosin staining of rat PTD sites show newly formed woven bone in the defect space in the PTD and PTD + liver injury rats (n = 3, Student's two-sided unpaired *t*-test). Scale bars, 200 µm. Representative micro-CT three-dimensional images (**b**) and bone volume/tissue volumes (BV/TV%) analyses (**c**) of defect sites of PTD and PTD + liver injury group, at 7 days after operation (n = 3; Student's two-sided unpaired *t*-test). The quantitation result were plotted as dot plots, showing the mean ± SEM of three independent experiments. ns = non-significant.

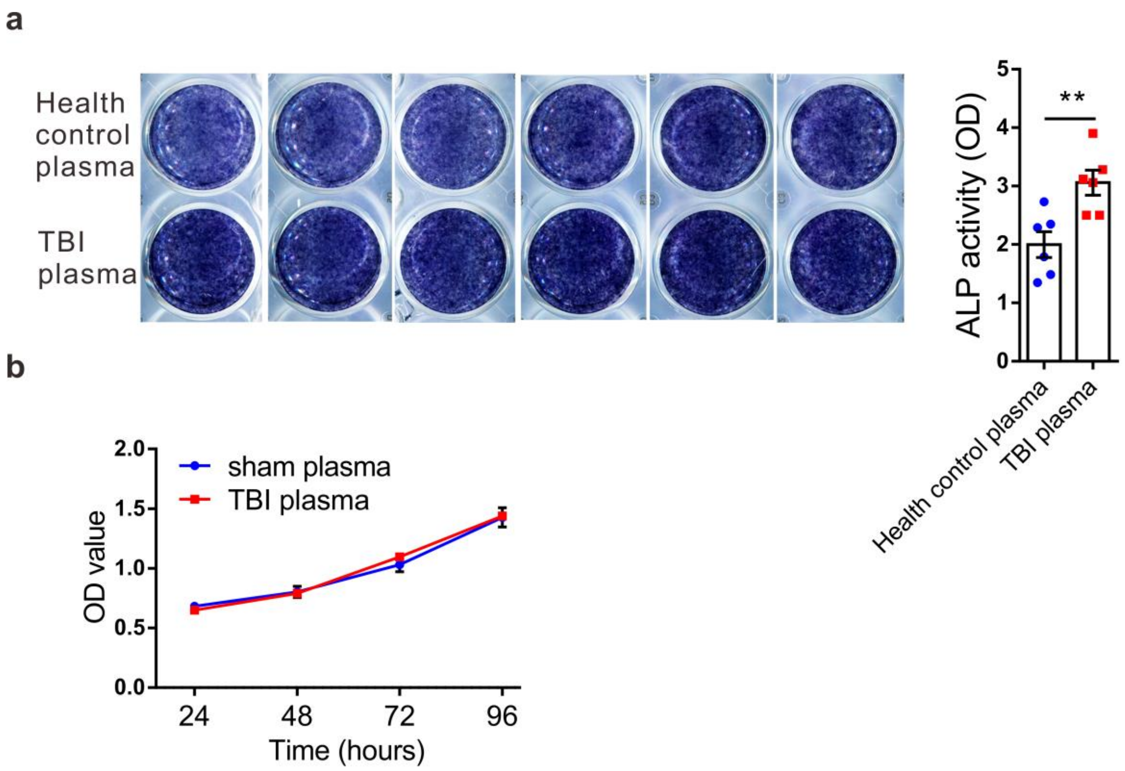

Supplementary Figure 5. Plasma from TBI patients contributes to TBI-stimulated osteogenesis, which is related to Figure 2.

(**a**) ALP staining and quantitation of MC3T3-E1 cells treated with plasma from TBI patients and healthy controls. (n = 3; Student's two-sided unpaired *t*-test). $P_{\text{(TBI plasma vs. Health control plasma)}}$=0.0064. The quantitative results were plotted as dot plots, showing the mean ± SEM of three independent experiments. (**b**) CCK-8 assays were performed to evaluate the effect of patient plasma on MC3T3-E1 proliferation (n = 6). The quantitation result were plotted as dot plots, showing the mean±SEM of three independent experiments. $^{**}P < 0.01$.

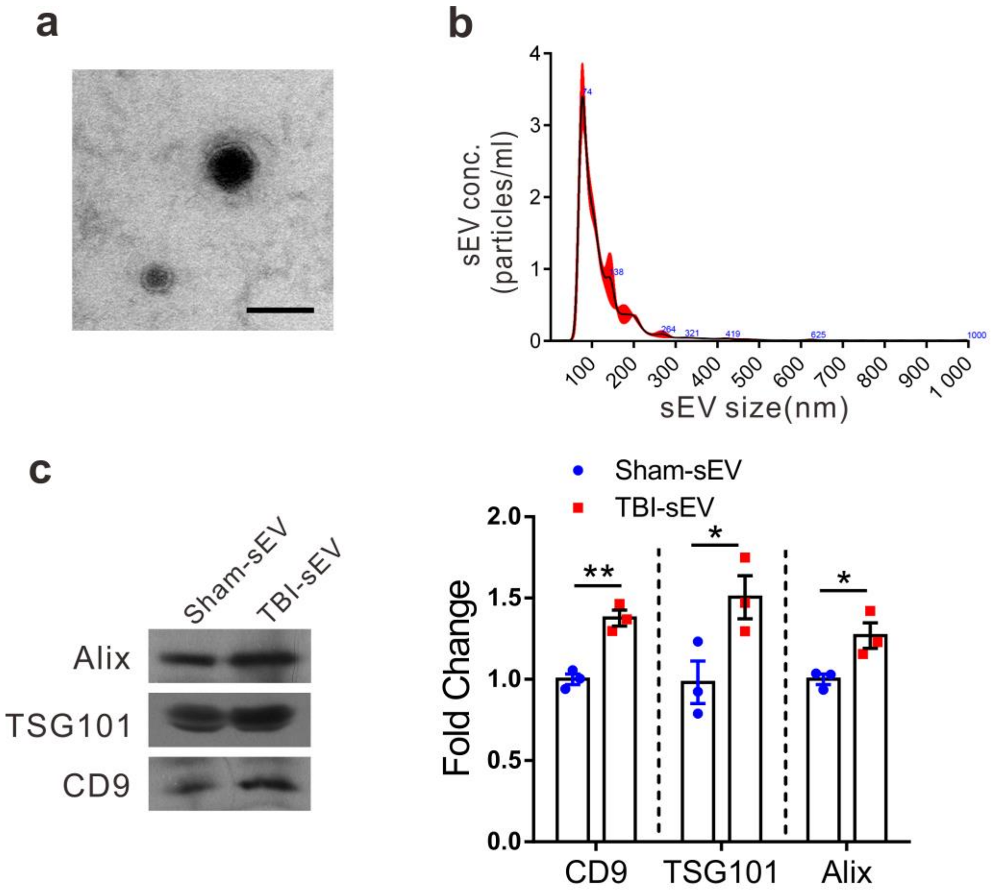

Supplementary Figure 6. The purified sEVs were verified by transmission electron microscopy (TEM), NanoSight analysis, and western blotting, which is related to Figure 2. (**a**) TEM image of sEVs isolated from the plasma of rats (n = 3). Scale bars: 100 nm. (**b**) NanoSight particle analysis displays the size distribution. (**c**) Western blot analysis of the typical sEVs markers Alix, TSG101, and CD9 of the sEVs isolated from the plasma (n = 3; Student's two-sided unpaired *t*-test). $P_{(CD9\ TBI\text{-}sEV\ vs.\ Sham\text{-}sEV)}=0.003$, $P_{(TSG101\ TBI\text{-}sEV\ vs.\ Sham\text{-}sEV)}=0.0484$, $P_{(Alix\ TBI\text{-}sEV\ vs.\ Sham\text{-}sEV)}=0.0339$.The quantitation result were plotted as dot plots, showing the mean±SEM of three independent experiments. $^{*}P < 0.05$, $^{**}P < 0.01$.

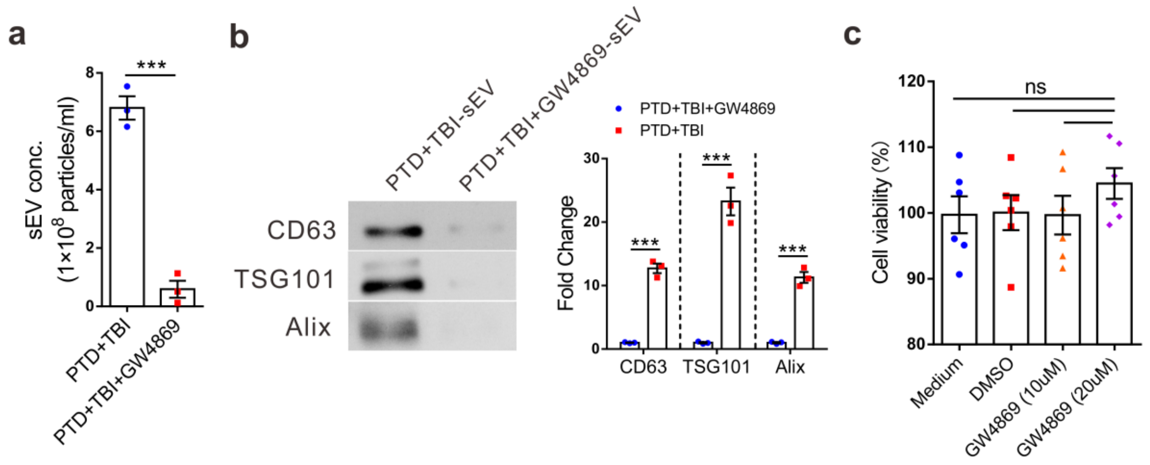

Supplementary Figure 7. The purified sEVs derived from the plasma of PTD+TBI and PTD+TBI+GW4869 groups were detected by NanoSight analysis and western blotting, which is related to Figure 2.

(**a**) The concentration of sEVs isolated from the plasma of rats was detected by NanoSight Analysis (n = 3, Student's two-sided unpaired *t*-test). $P_{(\text{PTD+TBI+GW4869 vs. PTD+TBI})}$=0.0002. (**b**) Western blot analysis of the typical sEVs markers CD63, TSG101, and Alix of the sEVs isolated from the PTD+TBI and PTD+TBI+GW4869 group plasma (n = 3, Student's two-sided unpaired *t*-test). $P_{(\text{CD63 PTD+TBI-sEV vs. PTD+TBI+GW4869-sEV})}$=0.0001, $P_{(\text{TSG101 PTD+TBI-sEV vs. PTD+TBI+GW4869-sEV})}$=0.0005, $P_{(\text{Alix PTD+TBI-sEV vs. PTD+TBI+GW4869-sEV})}$=0.0003. The quantitation result were plotted as dot plots, showing the mean ± SEM of three independent experiments. (**c**) Cytotoxicity of various concentrations of GW4869 evaluated via relative cell viability. No significant cytotoxicity was observed for treatment with various concentrations of GW4869. (n = 6; One-way analysis of variance with Turkey's multiple comparisons test was performed). The quantitation result were plotted as dot plots, showing the mean ± SEM of three independent experiments. $^{***}P < 0.001$; ns = no significant.

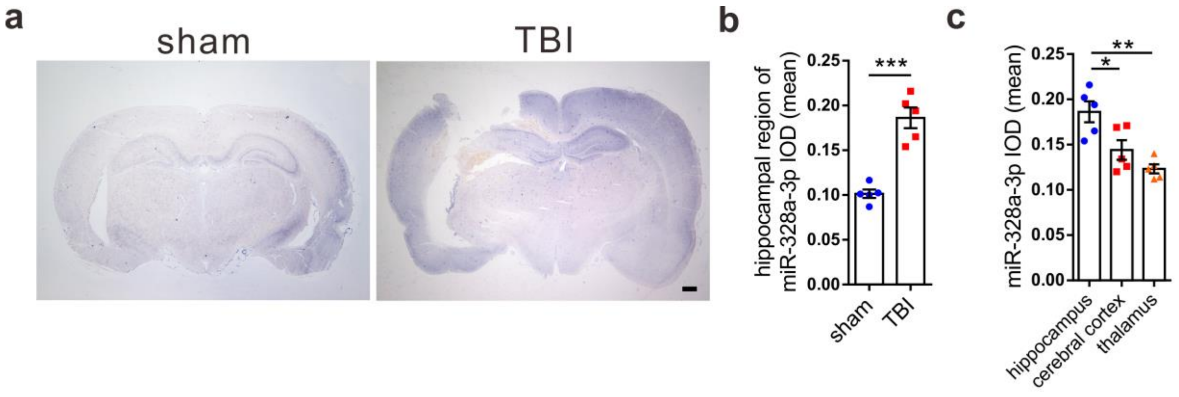

Supplementary Figure 8. In situ hybridization showed that expression of miR-328a-3p in brain tissue of rats increased significantly after TBI, especially in the hippocampus, which is related to Figure 5.

(**a**) In situ hybridization with a digoxigenin-labeled probe of miR-328a-3p in rat brain after TBI. Scale bar: 600 µm. The miR-328a-3p expression was quantified in the hippocampus (**b**) (n = 3, Student's two-sided unpaired *t*-test) and other regions (**c**) (n = 3; One-way analysis of variance with Turkey's multiple comparisons test was performed). $P_{(\text{TBI vs. sham})}$=0.0001, $P_{(\text{ hippocampus vs. cerebral cortex})}$=0.023, $P_{(\text{hippocampus vs. thalamus})}$=0.0015. The quantitation results were plotted as dot plots, showing the mean ± SEM of three independent experiments. $^{*}P < 0.05$, $^{**}P < 0.01$, $^{***}P < 0.001$.

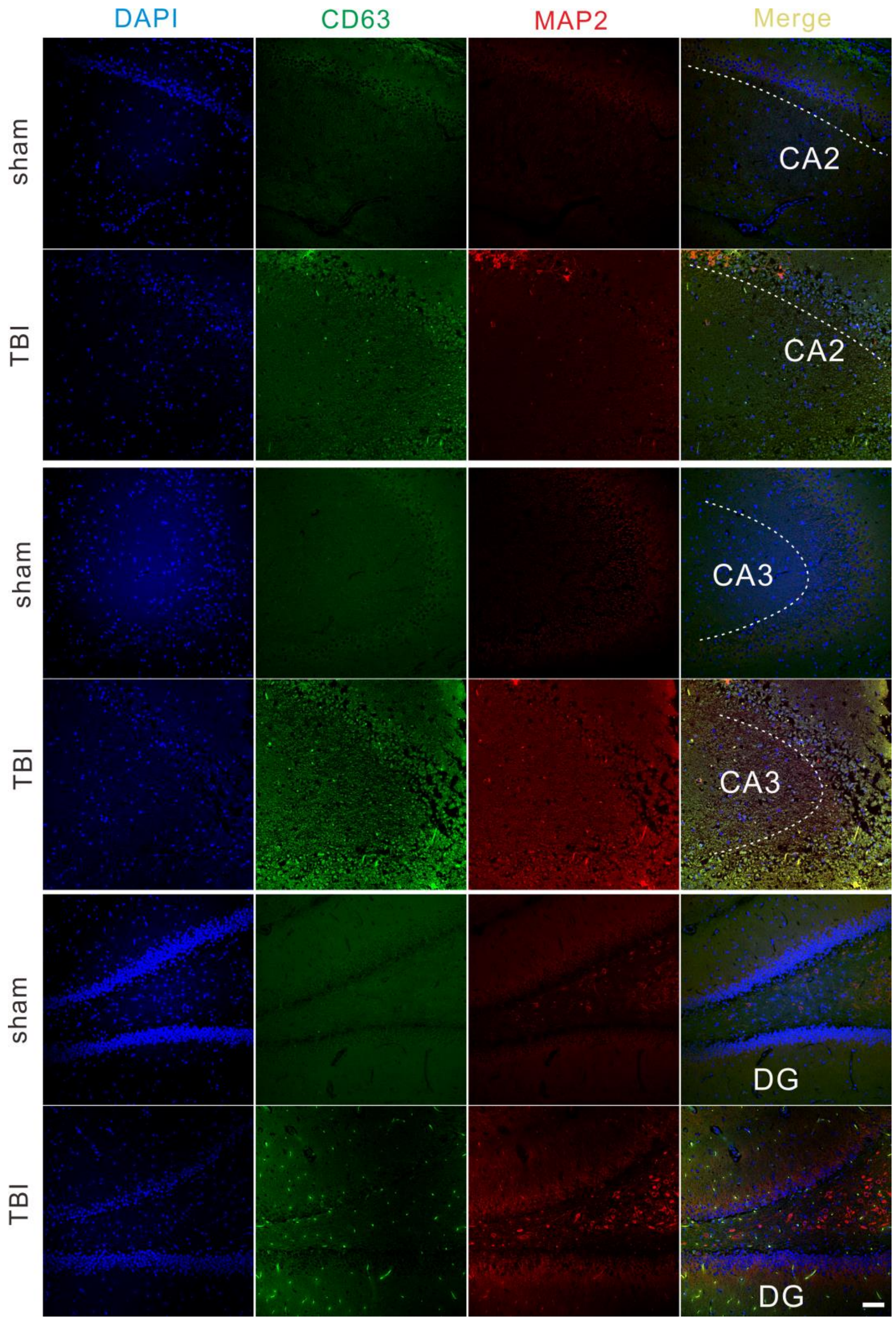

Supplementary Figure 9. Immunocytofluorescence staining of CD63 and MAP2 in different hippocampal regions, related to Figure 5.

Representative confocal images of immunocytofluorescence staining of CD63 (green), MAP2 (red), and DAPI (blue) staining in different hippocampal area (CA2, CA3, DG) of sham and TBI rats (n = 5; Student's two-sided unpaired *t*-test). Scale bars: 50 μm.

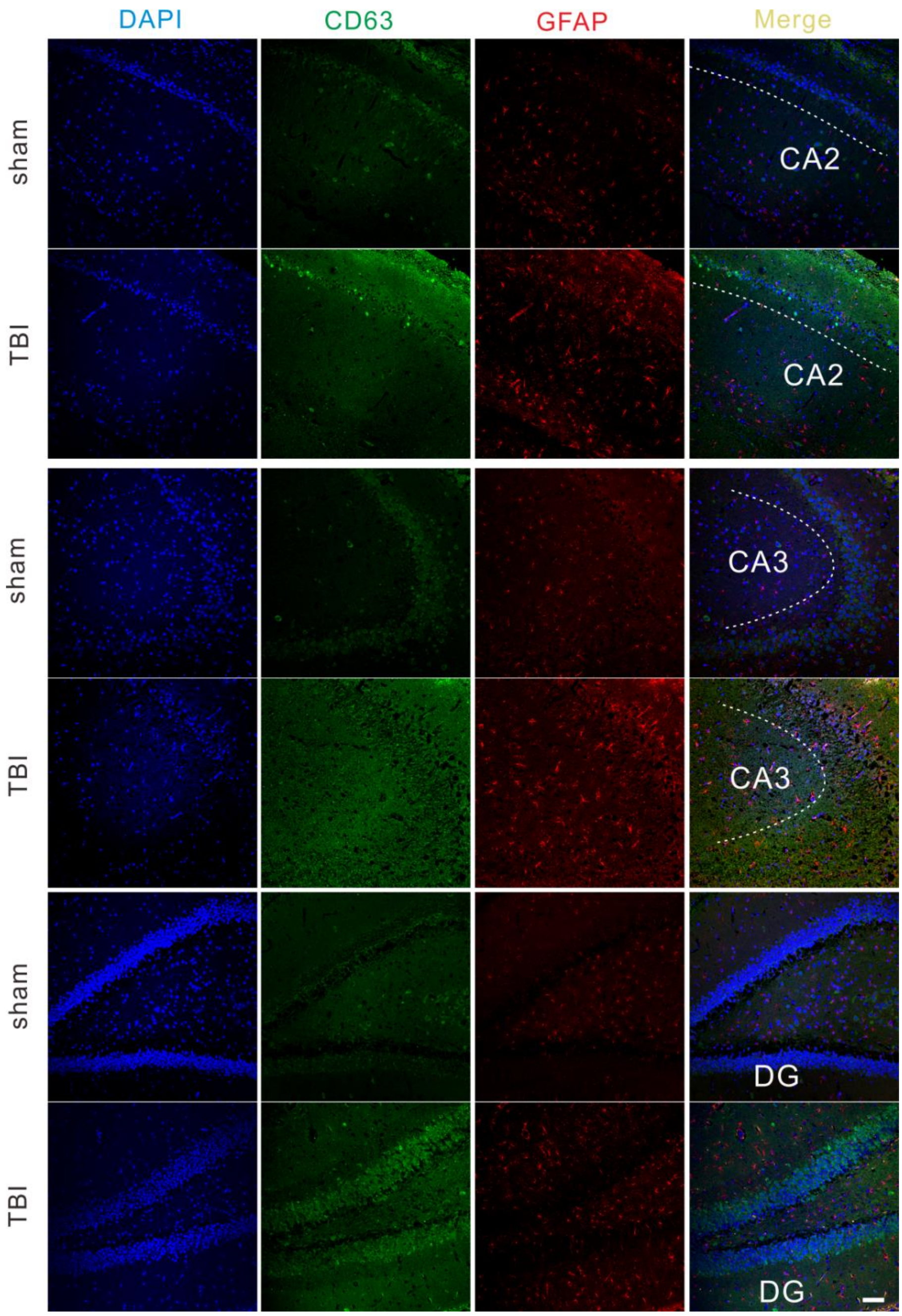

Supplementary Figure 10. Immunocytofluorescence staining of CD63 and GFAP in different hippocampal regions, related to Figure 5.

Representative confocal images of immunocytofluorescence staining of CD63 (green), astrocyte marker GFAP (red), and DAPI (blue) in different hippocampal area (CA2, CA3, DG) of sham and TBI rats (n = 5; Student's two-sided unpaired *t*-test). Scale bars: 50 µm.

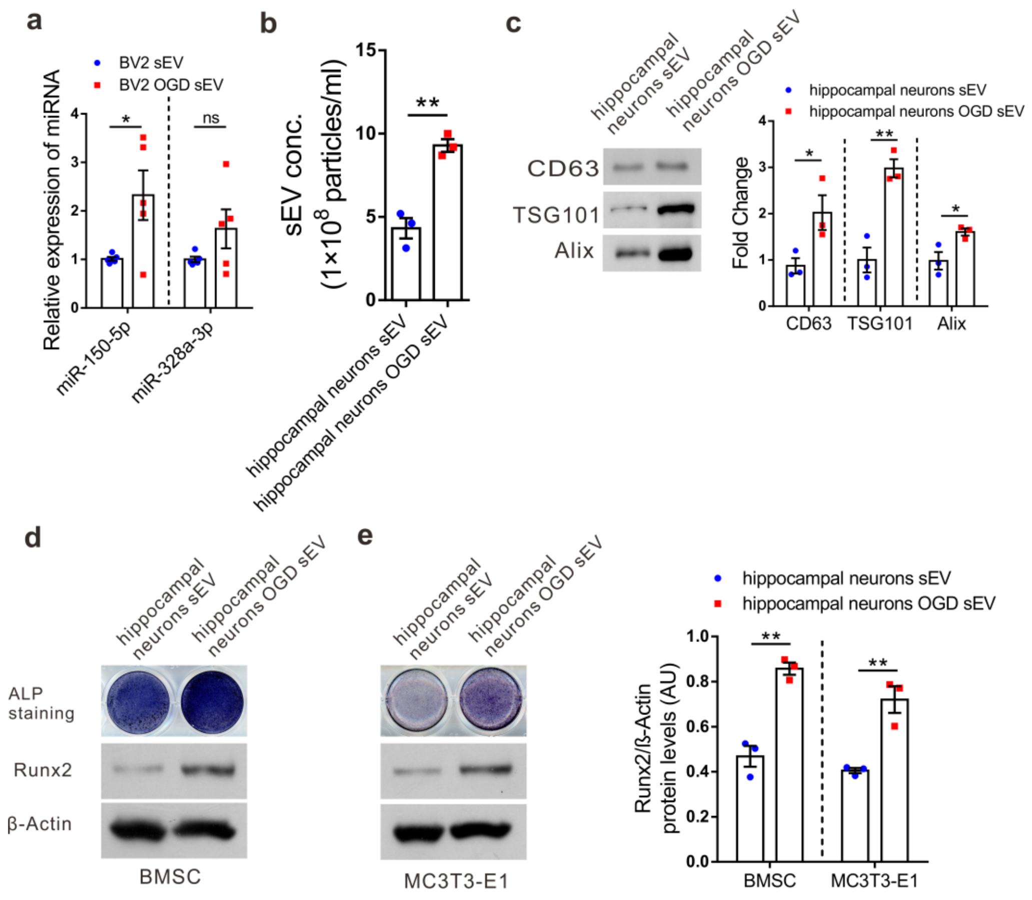

Supplementary Figure 11. The cross-talk between the injured neurons and osteoprogenitor cells through sEVs, related to Figure 5.

(**a**) Real-time quantitative PCR was used to detect the profile of miR-328a-3p and miR-150-5p in BV2 cell sEVs 24 h after OGD (oxygen-glucose deprivation) treatment (n = 5; Student's two-sided unpaired *t*-test). $P_{(\text{miR-150-5p BV2 OGD sEV vs. BV2 sEV})}$=0.034, $P_{(\text{miR-328a-3p BV2 OGD sEV vs. BV2 sEV})}$=0.1583. Concentration (**b**) and western blot analysis (**c**) of sEVs isolated from the primary hippocampal neurons or 24 h after OGD treatment (n = 3; Student's two-sided unpaired *t*-test). $P_{(\text{hippocampal neurons OGD sEV vs. hippocampal neurons sEV})}$=0.0023, $P_{(\text{CD63 hippocampal neurons OGD sEV vs. hippocampal neurons sEV})}$=0.0486, $P_{(\text{TSG101 hippocampal neurons OGD sEV vs. hippocampal neurons sEV})}$=0.004, $P_{(\text{Alix hippocampal neurons OGD sEV vs. hippocampal neurons sEV})}$=0.0382. The primary hippocampal neurons derived from younger than 12 h old SD rat pups were OGD or not and co-cultured with BMSC or MC3T3-E1

cells. ALP staining and western blotting of RUNX2 were performed of BMSC (**d**) and MC3T3-E1 cells (**e**) after co-cultured. β-actin served as a loading control (n = 3; Student's two-sided unpaired *t*-test). $P_{(\text{BMSC hippocampal neurons OGD sEV vs. hippocampal neurons sEV})}$=0.0019, $P_{(\text{MC3T3-E1 hippocampal neurons OGD sEV vs. hippocampal neurons sEV})}$=0.0065. The quantitation result were plotted as dot plots, showing the mean ± SEM of three independent experiments. $^{*}P < 0.05$, $^{**}P < 0.01$, ns, non-significant.

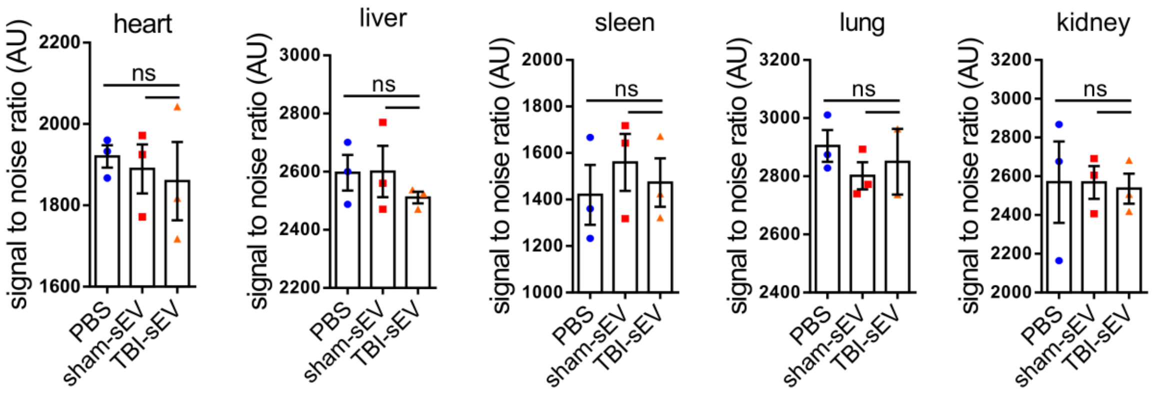

Supplementary Figure 12. PKH67-labeled sEVs purified from rats with TBI accumulated in the bone, but not in visceral organs, which is related to Figure 5.

Quantitation result of biophotonic images of the organ distribution of fluorescence signal in rats at 24 h after ICV brain infusion with purified PKH67-labelled sEVs isolated from rat plasma of sham, TBI groups or PBS only (n = 3; One-way analysis of variance with Turkey's multiple comparisons test was performed). The quantitation result were plotted as dot plots, showing the mean ± SEM of three independent experiments; ns, not significant.

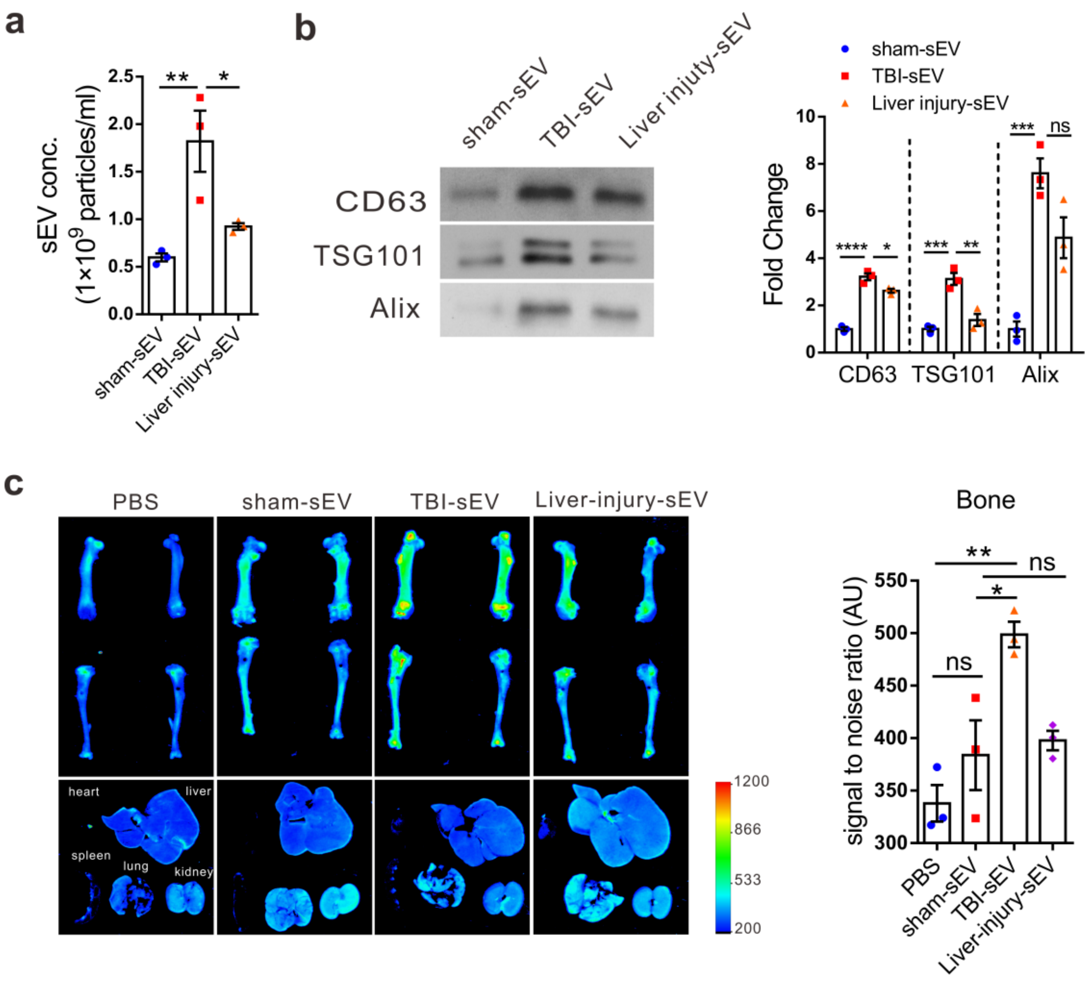

Supplementary Figure 13. Rat plasma sEVs increased after TBI.

(**a**) Concentration of sEVs isolated from the plasma of rats (n = 3; One-way analysis of variance with Turkey's multiple comparisons test was performed). $P_{(\text{sham-sEV vs. TBI-sEV})}=0.009$, $P_{(\text{TBI-sEV vs. Liver injury-sEV})}=0.0349$. (**b**) Western blot analysis of the typical sEVs markers CD63, TSG101, and Alix of sEVs isolated from the rat plasma (n = 3; One-way analysis of variance with Turkey's multiple comparisons test was performed). $P_{(\text{CD63 sham-sEV vs. TBI-sEV})}< 0.0001$, $P_{(\text{CD63 TBI-sEV vs. Liver injury-sEV})}=0.0186$, $P_{(\text{TSG101 sham-sEV vs. TBI-sEV})}=0.001$, $P_{(\text{TSG101 TBI-sEV vs. Liver injury-sEV})}=0.0028$, $P_{(\text{Alix sham-sEV vs. TBI-sEV})}=0.0009$, $P_{(\text{Alix TBI-sEV vs. Liver injury-sEV})}=0.0553$. (**c**) Representative biophotonic images of the organ distribution of fluorescence signal in rats at 6 h after intravenous injection with purified PKH67-labelled sEVs isolated from rat plasma of sham, TBI, liver injury groups, or PBS only groups (n = 3; One-way analysis of variance with Turkey's multiple comparisons test was performed). $P_{(\text{ PBS vs. sham-sEV})}=0.4301$, $P_{(\text{ PBS vs. TBI-sEV})}=0.0023$, $P_{(\text{ sham-sEV vs. TBI-sEV})}=0.0164$, $P_{(\text{ sham-sEV vs. Liver-injury-sEV})}=0.9592$. The quantitation result were plotted as dot plots, showing the mean±SEM of three independent experiments. $^{*}P < 0.05$, $^{**}P < 0.01$, $^{***}P < 0.001$, $^{****}P < 0.0001$.

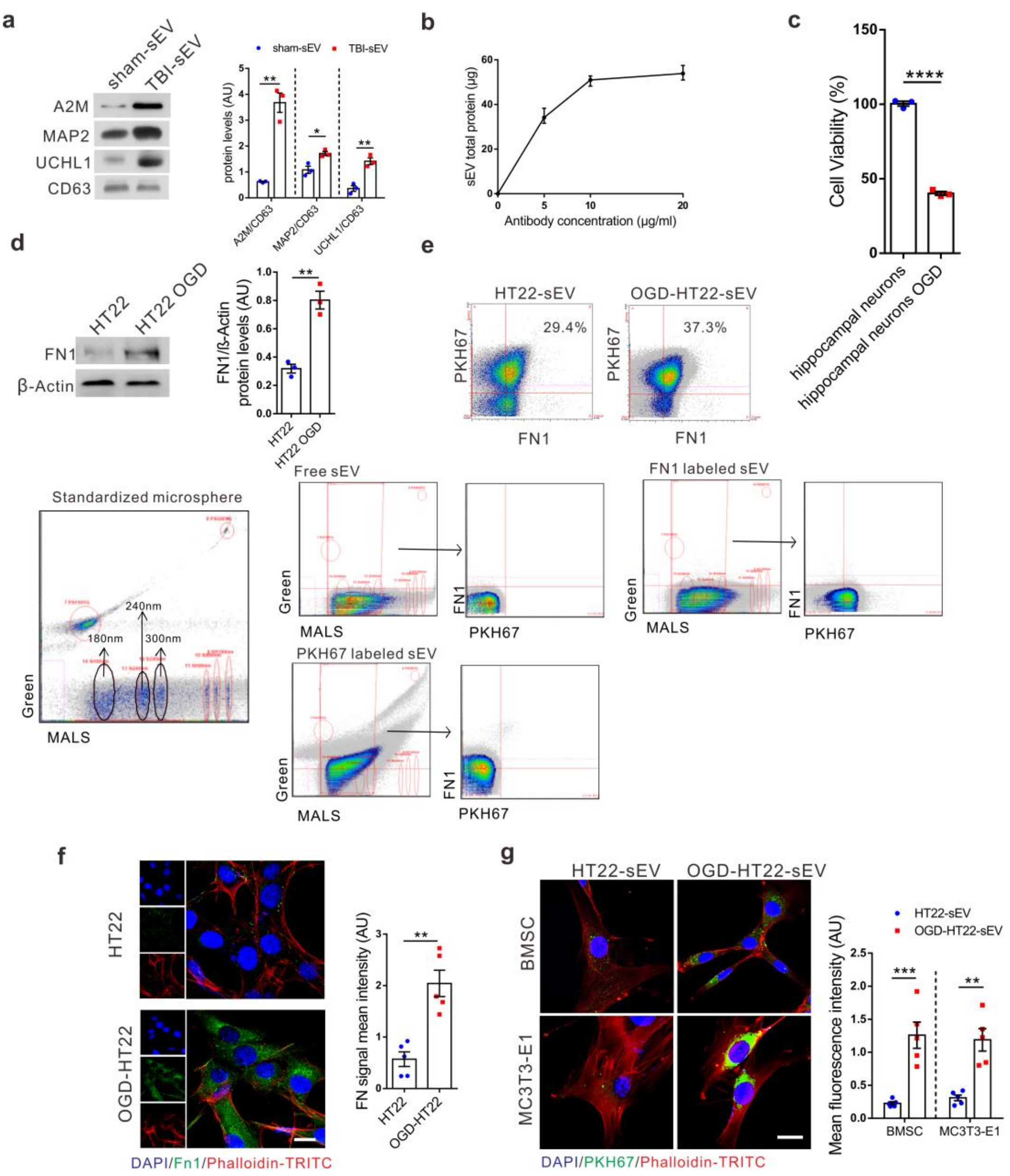

Supplementary Figure 14. Protein FN1 directs sEVs to target osteoprogenitors, which is related to Figure 6.

(**a**) Western blot analysis of A2M, MAP2 and UCHL1 in sEVs isolated from TBI and sham rat plasma (n = 3; Student's two-sided unpaired *t*-test). $P_{(\text{A2M/CD63 TBI-sEV vs. sham-sEV})}$=0.0012, $P_{(\text{MAP2/CD63 TBI-sEV vs. sham-sEV})}$=0.0167, $P_{(\text{UCHL1/CD63 TBI-sEV vs. sham-sEV})}$=0.0034. (**b**) An antibody titration curve was performed with rat sEVs (n = 4). (**c**) The cell viability was determined useing cell counting kit-8 assay. (n = 3; Student's two-sided unpaired *t*-test). $P_{(\text{hippocampal neurons OGD vs.}}$

$_{\text{hippocampal neurons)}}$< 0.0001. The quantitation result were plotted as dot plots, showing the mean ± SEM of three independent experiments. $^{****}P < 0.0001$. (**d**) Western blot analysis of FN1 in sEVs isolated from a mouse neuron cell line HT22, and 24 h after OGD treatment (n = 3; Student's two-sided unpaired *t*-test). $P_{(\text{HT22 OGD vs. HT22})}$=0.0023. (**e**) sEVs isolated from HT22 cells supernatant 24 h after OGD treatment were labeled with PKH67, antibodies FN1-PE, and analyzed by nanoscale Apogee flow cytometry. (**f**) Representative confocal images of immunocytofluorescence staining of F-actin (red) and FN1 (green), together with DAPI (blue) for nuclei in HT22 cells 24 h after OGD treatment or in the absence of OGD treatment (n = 5; Student's two-sided unpaired *t*-test). $P_{(\text{OGD-HT22 vs. HT22})}$=0.001. Scale bar: 20 μm. (**g**) BMSC and MC3T3-E1 cells were incubated with PKH67-labeled sEVs released by HT22 cells after OGD treatment and subjected to immunofluorescence of PKH-67-labeled sEVs (green), F-actin (red), and DAPI (blue) staining for nuclei (n = 5; Student's two-sided unpaired *t*-test). $P_{(\text{BMSC OGD-HT22-sEV vs. HT22-sEV})=}$ 0.0009, $P_{(\text{MC3T3-E1 OGD-HT22-sEV vs. HT22-sEV})=}$ 0.001. Scale bar, 20 μm. The quantitation result were plotted as dot plots, showing the mean±SEM of three independent experiments. $^{*}P < 0.05$, $^{**}P < 0.01$, $^{***}P < 0.001$, $^{****}P < 0.0001$.

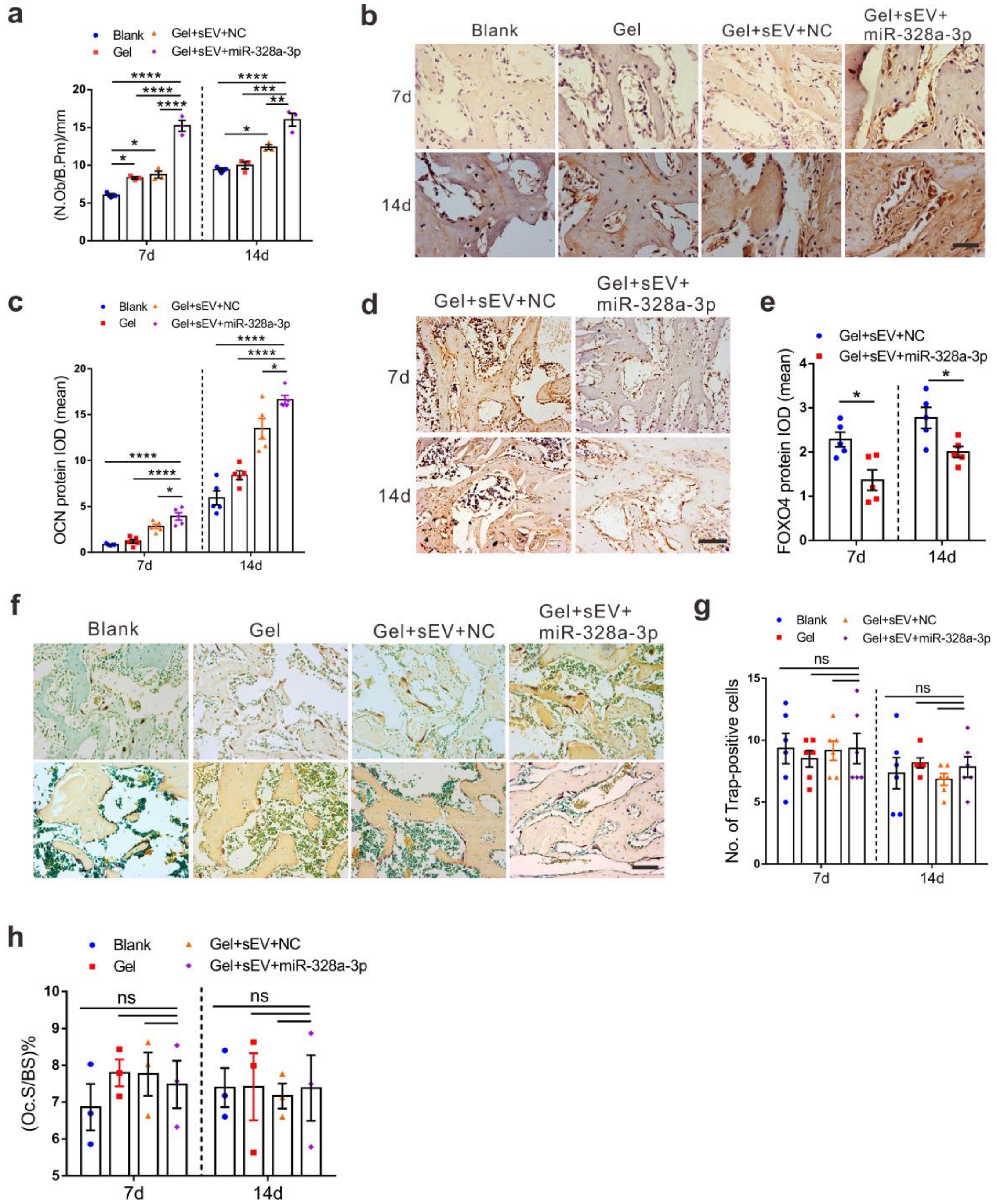

Supplementary Figure 15. Hydrogels carrying miR-328a-3p-containing sEVs efficiently promote bone formation and repair bone defects, which is related to Figure 7.

The numbers of osteoblasts (**a**) per bone surface area were assessed (n = 3; One-way analysis of variance with a Turkey's multiple comparisons test was performed). $P_{(\text{7d Blank vs. Gel})}$=0.0271, $P_{(\text{7d Blank vs. Gel+sEV+NC})}$=0.0107, $P_{(\text{7d Blank vs. Gel+sEV+miR-328a-3p})}$< 0.0001, $P_{(\text{7d Gel vs. Gel+sEV+miR-328a-3p})}$<

0.0001, $P_{(7d\ Gel+sEV+NC\ vs.\ Gel+sEV+miR\text{-}328a\text{-}3p)} < 0.0001$, $P_{(14d\ Blank\ vs.\ Gel+sEV+NC)}=0.0158$, $P_{(14d\ Blank\ vs.\ Gel+sEV+miR\text{-}328a\text{-}3p)} < 0.0001$, $P_{(14d\ Gel\ vs.\ Gel+sEV+miR\text{-}328a\text{-}3p)}=0.0002$, $P_{(14d\ Gel+sEV+NC\ vs.\ Gel+sEV+miR\text{-}328a\text{-}3p)}= 0.0051$. Immunohistochemical staining of OCN (**b**), FOXO4 (**d**), and quantitation of OCN and FOXO4 staining (**c**), (**e**) with Image J (n = 5; One-way analysis of variance with a Turkey's multiple comparisons test was performed).

$P_{(OCN\ 7d\ Blank\ vs.\ Gel+sEV+miR\text{-}328a\text{-}3p)} < 0.0001$, $P_{(OCN\ 7d\ Gel\ vs.\ Gel+sEV+miR\text{-}328a\text{-}3p)} < 0.0001$, $P_{(OCN\ 7d\ Gel+sEV+NC\ vs.\ Gel+sEV+miR\text{-}328a\text{-}3p)}=0.0403$, $P_{(OCN\ 14d\ Blank\ vs.\ Gel+sEV+miR\text{-}328a\text{-}3p)} < 0.0001$, $P_{(OCN\ 14d\ Gel\ vs.\ Gel+sEV+miR\text{-}328a\text{-}3p)} < 0.0001$, $P_{(OCN\ 14d\ Gel+sEV+NC\ vs.\ Gel+sEV+miR\text{-}328a\text{-}3p)}=0.0417$, $P_{(FOXO4\ 7d\ Gel+sEV+miR\text{-}328a\text{-}3p\ vs.\ Gel+sEV+NC)}=0.0121$, $P_{(FOXO4\ 14d\ Gel+sEV+miR\text{-}328a\text{-}3p\ vs.\ Gel+sEV+NC)}=0.0213$. Scale bars: 40 µm. Representativeg images of TRAP stainin (**f**) and quantitation of TRAP-positive cells (**g**) with Image J at 7 and 14 days after surgery (n = 6; One-way analysis of variance with a Turkey's multiple comparisons test was performed). Scale bars, 50 µm. The numbers of osteoclasts (**h**) per bone surface area were assessed (n = 3; One-way analysis of variance with a Turkey's multiple comparisons test was performed). The quantitation result were plotted as dot plots, showing the mean ± SEM of three independent experiments. $^{*}P < 0.05$, $^{****}P < 0.0001$; ns non-significant.

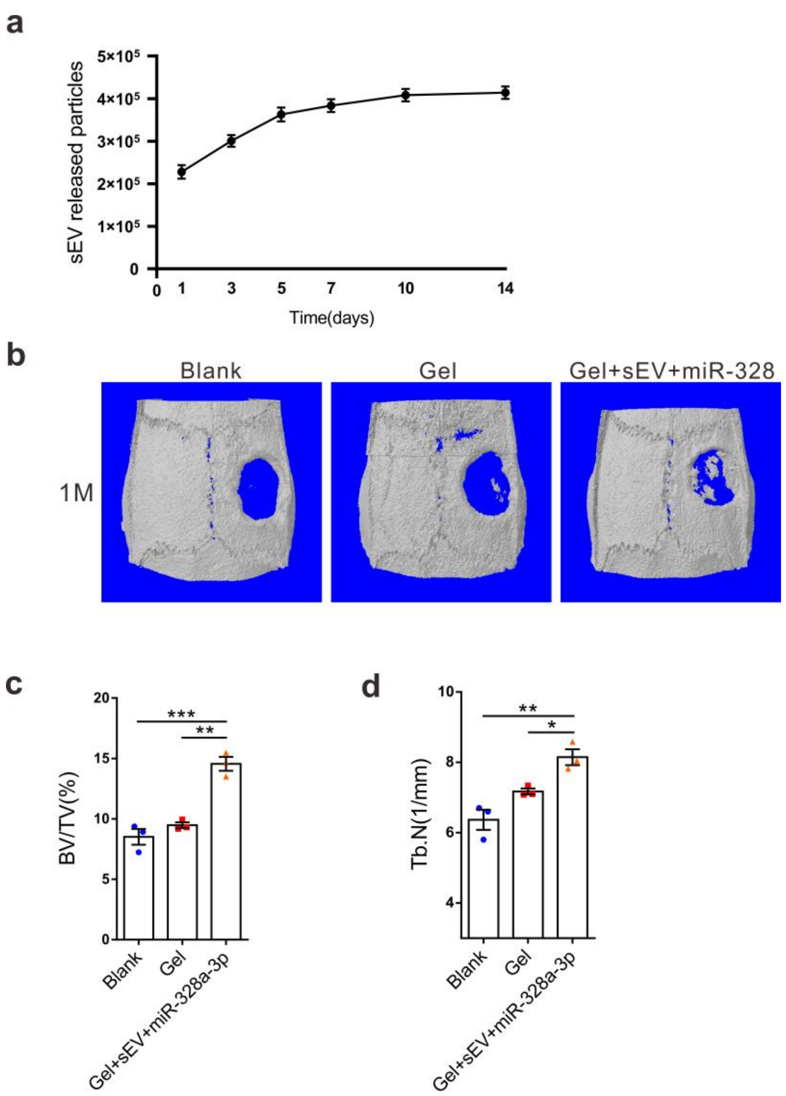

Supplementary Figure 16. Hydrogels carrying miR-328a-3p-containing sEVs efficiently promote bone formation in a calvarial defect model, which is related to Figure 7.

(**a**) sEVs release kinetics from hydrogels (n=6). Three-dimensional micro-computed tomography (CT) image (**b**) and quantitative data of BV/TV% (**c**) and Tb.n (**d**) were obtained from micro-CT analysis at 1 month after the calvarial defects of rats treated with/without hydrogels encapsulated with sEVs miR-328a-3p (n = 3. One-way analysis of variance with Turkey's multiple comparisons test was performed). $P_{(BV/TV\ Blank\ vs.\ Gel+sEV+miR\text{-}328a\text{-}3p)}$=0.0004, $P_{(BV/TV\ Gel\ vs.\ Gel+sEV+miR\text{-}328a\text{-}3p)}$=0.0011, $P_{(Tb.n\ Blank\ vs.\ Gel+sEV+miR\text{-}328a\text{-}3p)}$=0.0027, $P_{(Tb.n\ Gel\ vs.\ Gel+sEV+miR\text{-}328a\text{-}3p)}$=0.0427. The quantitation result were plotted as dot plots, showing the mean ± SEM of three independent experiments. $^{*}P < 0.05$, $^{**}P < 0.01$, $^{***}P < 0.001$.

Supplementary tables

Supplementary table 1 Baseline clinical parameters of patients with fracture alone and concomitant TBI, which is related to Figure 1.

Patient	Diagosis	Surgical Operation	GCS Score	AO/OTA Fracture Classification	Location of TBI and Fracture
1	Proximal Femural Fracture (Left)	internal fixation with intramedullary nail	E4V5M6=15	31-A1	
2	Femural Fracture (Right)	internal fixation with plate	E4V5M6=15	32-B2	
3	Proximal Femural Fracture (Left)	internal fixation with intramedullary nail	E4V5M6=15	31-A3	
4	Femural Fracture (Left)	internal fixation with intramedullary nail	E4V5M6=15	31-B2	
5	Proximal Femural Fracture (Right)	internal fixation with intramedullary nail	E4V5M6=15	31-A1	
6	Proximal Femural Fracture (Right)	internal fixation with intramedullary nail	E4V5M6=15	31-A3	
7	Femural Fracture (Right)	internal fixation with plate	E4V5M6=15	32-C1	
8	Femural Fracture (Right)	internal fixation with plate	E4V5M6=15	32-A2	
9	Femural Fracture (Right)	internal fixation with plate	E4V5M6=15	31-A3	
10	Femural Fracture (Left)	internal fixation with plate	E4V5M6=15	32-B1	
11	Proximal Femural Fracture (Left)	internal fixation with plate	E4V5M6=15	31-B3	
12	Femural Fracture (Left)	internal fixation with intramedullary nail	E4V5M6=15	33-C1	
13	Femural Fracture (Left) concomitant with Occipital and Skull Base Fractures	internal fixation with intramedullary nail	E2V2M4=8	32-B2	Ipsilateral OR contralateral
14	Proximal Femural Fracture (Left) concomitant with Maxillary and Mandible Fractures (Right)	internal fixation with intramedullary nail	E3V2M3=8	31-A3	Contralateral
15	Proximal Femural Fracture (Right) concomitant with Occipital Fracture (Right)	internal fixation with intramedullary nail	E2V3M3=8	31-C2	Ipsilateral
16	Proximal Femural Fracture (Right) concomitant with Subarachnoid	internal fixation with plate	E2V1M4=7	31-B1	Ipsilateral OR

	Hemorrhage				contralateral
17	Proximal Femural Fracture (Right) concomitant with Subarachnoid Hemorrhage and Occipital Fracture (Right)	internal fixation with plate	E2V1M3=6	31-A2	Contralateral
18	Femural Fracture (Left) concomitant with Frontal Hemorrhage (Left)	internal fixation with intramedullary nail	E2V1M3=6	32-A2	Ipsilateral
19	Proximal Femural Fracture (Left) concomitant with Bilateral Frontotemporal Brain Contusion and Laceration	internal fixation with plate	E2V2M4=8	31-A3	Ipsilateral OR contralateral
20	Femural Fracture (Left) concomitant with Frontal Hemorrhage (Left)	internal fixation with intramedullary nail	E2V2M3=7	32-B2	Ipsilateral
21	Proximal Femural Fracture (Left) concomitant with Hypoxic-ischemic Encephalopathy	internal fixation with plate	E3V2M2=7	31-A2	Ipsilateral OR contralateral
22	Femural Fracture (Right) concomitant with Skull Base Fracture and Subarachnoid Hemorrhage	internal fixation with plate	E3V3M2=8	33-A2	Ipsilateral OR contralateral
23	Femural Fracture (Right) concomitant with Parietal and Temporal Fractures (Left)	internal fixation with intramedullary nail	E2V2M4=8	31-A2	Contralateral
24	Femural Fracture (Right) concomitant with Frontoparietal Subdural Hematoma (Left)	internal fixation with intramedullary nail	E3V2M3=8	32-B2	Contralateral

Supplementary table 2 MiRNAs examined in the plasma sEVs of TBI vs. sham group, which is related to Figure 3.

miRNA name	Sequence	Accession	24h_count	72h_count	Sham_count	24h_Tpm	72h_Tpm	Sham_Tpm	24h	72h	Sham	24h vs sham log2FC	Pvalue	72h vs sham log2FC	72h vs sham Pvalue
rno-miR-877	GUAGAGGAGAUGGCGCAGGG	MIMAT0005285	20	14	14	56.030442783	68.37983021	18.640794981	56.030442783	68.37983021	18.640794981	1.587747505	0.011048418	1.875107452	0.013197567
rno-miR-872-5p	AAGGUUACUUGUUAGUUCAGG	MIMAT0005282	68	37	350	190.506360516	180.719233013	466.006327424	190.506360516	180.719233013	466.006327424	-1.290510378	2.41839E-07	-1.366599491	4.55731E-07
rno-miR-674-3p	CACAGCUCCCAUCUCAGAACAA	MIMAT0005330	32	19	14	89.648708452	92.800087828	18.640794981	89.648708452	92.800087828	18.640794981	2.26581941	4.27605E-05	2.315662782	0.000352577
rno-miR-671	UCCGGUUCUCAGGGCUCCACC	MIMAT0005326	81	73	39	226.925434561	356.552341915	51.925993576	226.925434561	356.552341915	51.925993576	2.127689499	1.00842E-08	2.779585058	7.7041E-13
rno-miR-340-5p	UUAUAAAGCAAUGAGACUGAUU	MIMAT0004650	9	7	78	25.212985489	34.187324371	103.853218705	25.212985489	34.187324371	103.853218705	-2.042307066	8.76189E-06	-1.603012509	0.000993175
rno-miR-328a-3p	CUGGCCCUCUCUGCCCUUCCGU	MIMAT0000564	882	545	728	2470.961084575	2661.927254089	969.293013256	2470.961084575	2661.927254089	969.293013256	1.350067532	3.51025E-08	1.457466388	1.77179E-08
rno-miR-326-3p	CCUCUGGGCCCUUCCUCCAGU	MIMAT0000560	60	64	43	168.091328348	312.592769323	57.252462808	168.091328348	312.592769323	57.252462808	1.55383564	9.76363E-05	2.448874748	3.18276E-10
rno-miR-322-5p	CAGCAGCAAUUCAUGUUUUGGA	MIMAT0001619	13	3	64	36.419074045	14.653190864	85.212423725	36.419074045	14.653190864	85.212423725	-1.226369544	0.004052845	-2.539848928	0.000134587
rno-miR-32-5p	UAUUGCACAUUACUAAGUUGCA	MIMAT0000811	1	0	18	2.800808376	0	23.966032659	2.800808376	0	23.966032659	-3.097075919	0.00572059		0.007880255
rno-miR-219a-1-3p	AGAGUUGCGUCUGGACGUCCCG	MIMAT0004741	25	15	10	70.037339715	73.265954321	13.314325749	70.037339715	73.265954321	13.314325749	2.395144915	9.82733E-05	2.460163583	0.000672116
rno-miR-218a-5p	UUGUGCUUGAUCUAACCAUGU	MIMAT0000888	12	6	65	33.61826567	29.306381728	86.543733144	33.61826567	29.306381728	86.543733144	-1.364184055	0.001961088	-1.562214495	0.003778445
rno-miR-212-3p	UAACAGUCUCCAGUCACGGCCA	MIMAT0000883	76	36	43	212.918537629	175.833108902	57.252462808	212.918537629	175.833108902	57.252462808	1.894891903	5.67815E-07	1.618797092	0.000730758
rno-miR-205	UCCUUCAUUCCACCGGAGUCUGU	MIMAT0000878	26	32	17	72.841003145	156.298975395	22.634723239	72.841003145	156.298975395	22.634723239	1.686213124	0.002421522	2.787698749	2.26803E-07
rno-miR-203a-3p	GUGAAAUGUUUAGGACCACUAG	MIMAT0000876	11	8	104	30.817457294	39.073448482	138.470958274	30.817457294	39.073448482	138.470958274	-2.167763692	1.99595E-07	-1.825322936	6.4902E-05
rno-miR-194-5p	UGUAACAGCAACUCCAUGUGGA	MIMAT0000869	6	1	43	16.810560362	4.886124111	57.252462808	16.810560362	4.886124111	57.252462808	-1.767969938	0.002545854	-3.55057534	0.000165623
rno-miR-150-5p	UCUCCCAACCCUUGUACCAGUG	MIMAT0000853	5295	3334	4316	14834.174642685	16284.145930921	5746.521368846	14834.174642685	16284.145930921	5746.521368846	1.368163863	4.45971E-09	1.502707259	5.24401E-10
rno-miR-141-3p	UAACACUGUCUGGUAAAGAUGG	MIMAT0000846	49	37	413	137.276726109	180.719233013	549.88744173	137.276726109	180.719233013	549.88744173	-2.002049287	9.96203E-14	-1.605386286	4.79536E-09
rno-miR-139-5p	UCUACAGUGCACGUGUCUCCAG	MIMAT0000845	87	63	57	243.733139869	307.706645212	75.892026235	243.733139869	307.706645212	75.892026235	1.683282206	2.66082E-06	2.019535382	1.93007E-07
rno-miR-1247-5p	ACCCGUCCCGUUCGUCCCCGGA	MIMAT0035721	44	33	30	123.266974122	161.179918038	39.942977247	123.266974122	161.179918038	39.942977247	1.625772546	0.000267908	2.012658228	4.25715E-05
rno-miR-122-5p	UGGAGUGUGACAAUGGUGUUUG	MIMAT0000827	107	34	24	299.766437706	166.066042149	31.955120729	299.766437706	166.066042149	31.955120729	3.229719823	2.11001E-17	2.377638052	2.8034E-06
rno-miR-101a-3p	UACAGUACUGUGAUAACUGAA	MIMAT0000823	74	61	636	207.314065824	297.939578459	846.800231124	207.314065824	297.939578459	846.800231124	-2.03020366	3.08203E-16	-1.507001878	5.02803E-10
