# Supplementary material for: Damaged brain accelerates bone healing by releasing small extracellular vesicles that target osteoprogenitors
Source: Nat Commun. 2021 Oct 15;12:6043. doi: 10.1038/s41467-021-26302-y (PMC8519911; doi:10.1038/s41467-021-26302-y)
Supplement: Supplementary file 2 — Reporting Summary [file 41467_2021_26302_MOESM2_ESM.pdf]

nature research

Corresponding author(s): Qiancheng Song, PhD
Xiaochun Bai, PhD

Last updated by author(s): 2021/07/18

Reporting Summary

Nature Research wishes to improve the reproducibility of the work that we publish. This form provides structure for consistency and transparency in reporting. For further information on Nature Research policies, see Authors & Referees and the Editorial Policy Checklist .

Statistics

For all statistical analyses, confirm that the following items are present in the figure legend, table legend, main text, or Methods section.

n/a	Confirmed	
☐	☒	The exact sample size (n) for each experimental group/condition, given as a discrete number and unit of measurement
☐	☒	A statement on whether measurements were taken from distinct samples or whether the same sample was measured repeatedly
☐	☒	The statistical test(s) used AND whether they are one- or two-sided<br>*Only common tests should be described solely by name; describe more complex techniques in the Methods section.*
☒	☐	A description of all covariates tested
☒	☐	A description of any assumptions or corrections, such as tests of normality and adjustment for multiple comparisons
☐	☒	A full description of the statistical parameters including central tendency (e.g. means) or other basic estimates (e.g. regression coefficient) AND variation (e.g. standard deviation) or associated estimates of uncertainty (e.g. confidence intervals)
☐	☒	For null hypothesis testing, the test statistic (e.g. F, t, r) with confidence intervals, effect sizes, degrees of freedom and P value noted<br>*Give P values as exact values whenever suitable.*
☒	☐	For Bayesian analysis, information on the choice of priors and Markov chain Monte Carlo settings
☒	☐	For hierarchical and complex designs, identification of the appropriate level for tests and full reporting of outcomes
☒	☐	Estimates of effect sizes (e.g. Cohen's d, Pearson's r), indicating how they were calculated

Our web collection on statistics for biologists contains articles on many of the points above.

Software and code

Policy information about availability of computer code

Data collection	WPS Office 11.1 perform data collection.
Data analysis	WPS Office 11.1 and Graphpad Prism 6.0 were used to perform statistical analyses. Proteomic analyses of exosomal proteins were processed using Proteome Discoverer (Thermo Fisher Scientific, Version 2.4) against Uniprot Rattus norvegicus database (version 20200617; 35,779 sequences). Image Pro Plus 6.0 software (Media Cybernetics, MD, USA) was used to measure the percentage of bone matrix within the callus. NanoSight NS500 (Malvern, Westborough, MA, USA) was used to measure the size and the concentration of the isolated sEVs.

For manuscripts utilizing custom algorithms or software that are central to the research but not yet described in published literature, software must be made available to editors/reviewers. We strongly encourage code deposition in a community repository (e.g. GitHub). See the Nature Research guidelines for submitting code & software for further information.

Data

Policy information about availability of data

All manuscripts must include a data availability statement. This statement should provide the following information, where applicable:

- Accession codes, unique identifiers, or web links for publicly available datasets
- A list of figures that have associated raw data
- A description of any restrictions on data availability

The miRNA-seq Illumina reads for all samples data generated in this study have been deposited into the Sequence Read Archive at the National Center for Biotechnology Information database under accession code PRJNA670580 (http://www.ncbi.nlm.nih.gov/sra). The mass spectrometry proteomics data used in this study are available in the PRIDE database under accession code PXD022126 (http://www.proteomexchange.org). All other relevant data are available from the authors upon reasonable request. Source data are provided with this paper.

Field-specific reporting

Please select the one below that is the best fit for your research. If you are not sure, read the appropriate sections before making your selection.

[x] Life sciences [ ] Behavioural & social sciences [ ] Ecological, evolutionary & environmental sciences

For a reference copy of the document with all sections, see nature.com/documents/nr-reporting-summary-flat.pdf

Life sciences study design

All studies must disclose on these points even when the disclosure is negative.

Sample size	Sample sizes were chosen based on previous experience with the animal models or following convention of the methods. All sample sizes are listed in detail in the figure legends and main text. For gene and protein expression level analysis by qPCR and Western Blot, combining a technical duplicate (intra-sample variability) with at least a biological triplicate (inter-experiment variability) was applied to detect a prespecified effect size.
Data exclusions	No data were excluded from the analyses.
Replication	All data presented were from biological replicates. All attempts at replication were successful. For some findings, several methods have been used to verify the results. Data described in this manuscript were reliably reproduced.
Randomization	The animals were randomly assigned to experimental groups.
Blinding	Investigators were not blinded for group allocation for practical reasons. However, the experimenter were blinded during data collection and analyses.

Reporting for specific materials, systems and methods

We require information from authors about some types of materials, experimental systems and methods used in many studies. Here, indicate whether each material, system or method listed is relevant to your study. If you are not sure if a list item applies to your research, read the appropriate section before selecting a response.

Materials & experimental systems

n/a	Involved in the study	
[ ]	[x]	Antibodies
[ ]	[x]	Eukaryotic cell lines
[x]	[ ]	Palaeontology
[ ]	[x]	Animals and other organisms
[ ]	[x]	Human research participants
[x]	[ ]	Clinical data

Methods

n/a	Involved in the study	
[x]	[ ]	ChIP-seq
[ ]	[x]	Flow cytometry
[x]	[ ]	MRI-based neuroimaging

Antibodies

Antibodies used

The following primary antibodies were used for western blot
TSG101 (cat# ab83, dilution 1:1,000) from Abcam (Cambridge, UK)
CD63 (cat# ab193349, dilution 1:1,000) from Abcam (Cambridge, UK)
CD9 (cat# ab223052, dilution 1:1,000) from Abcam (Cambridge, UK)
Alix (cat# ab76608, dilution 1:1,000) from Abcam (Cambridge, UK)
Runx2 (cat# 12556, dilution 1:1,000) from Cell Signaling Technology (MA, USA)
FN1 (cat# 15613-1-AP, dilution 1:1,000) from Proteintech (Wuhan, China)
FOXO4 (cat# 21535-1-AP, dilution 1:1000) from Proteintech (Wuhan, China)
CBL (cat# A7881, dilution 1:1000) from ABclonal (Wuhan, China)
β-Actin (cat# 4970S, dilution 1:4000) from Cell Signaling Technology (MA, USA)
GAPDH (cat# 2118, 1:5000) from Cell Signaling Technology (MA, USA)
UCHL1 (Cell Signaling Technology, cat#13179, 1:1000)
MAP2 (Cell Signaling Technology, cat#: 4542S, 1:1000).
Secondary antibodies for western blot
anti-rabbit IgG, (cat# 7074, dilution 1:2000) from Cell Signaling Technology (MA, USA)
anti-mouse IgG, (cat# A9044, dilution 1:2000) from Sigma Aldrich (MO, USA)

The following primary antibodies were used for immunhistological staining:

	osteocalcin (cat#ab13420, dilution 1:200) from Abcam (Cambridge, UK)<br>FoxO4 (cat#A21535-1-AP, dilution 1:100) from Proteintech (Wuhan, China)<br>Cbl (cat#A7881, dilution 1:100) from ABclonal (Wuhan, China)<br>Secondary antibodies for immunhistological staining:<br>anti-rabbit IgG (cat# 7074, dilution 1:200) from Cell Signaling Technology (MA, USA)<br>anti-mouse IgG (cat# A9044, dilution 1:200) from Sigma Aldrich (MO, USA)<br><br>The following primary antibodies were used for immunofluorescence staining:<br>CD63 (cat#ab193349, dilution 1:50) from Abcam (Cambridge, UK)<br>FN1 (cat#15613-1-AP, dilution 1:100) from Proteintech (Wuhan, China)<br>GFAP (cat#MAB360, dilution 1:100) from Merck (Darmstadt, Germany)<br>A2M (cat#A1573, dilution 1:100) from ABclonal (Wuhan, China)<br>MAP2 (cat#4542S, dilution 1:100) from Cell Signaling Technology (MA, USA)<br><br>Secondary antibodies for immunofluorescence staining<br>Goat anti-rabbit Alexa Fluor 488 (cat#A-11008, dilution 1:500) from Invitrogen (MA, USA)<br>Goat anti-mouse Alexa Fluor 594 (cat#A-11032, dilution 1:500) from Invitrogen (MA, USA)
Validation	TSG101 (cat#ab83) from Abcam (Cambridge, UK) Validated by the company and by users (cited 137 times)<br>CD63 (cat#ab193349) from Abcam (Cambridge, UK) Validated by the company and by users (cited 28 times)<br>CD9 (cat#ab223052) from Abcam (Cambridge, UK) Validated by the company and by users (cited 4 times)<br>Alix (cat#ab76608) from Abcam (Cambridge, UK) Validated by the company and by users (cited 5 times)<br>Runx2 (cat#12556) from Cell Signaling Technology (MA, USA) Validated by the company and by users (cited 72 times)<br>FN1 (cat#15613-1-AP) from Proteintech (Wuhan, China) Validated by the company and by users (cited 140 times)<br>FOXO4 (cat#21535-1-AP) from Proteintech (Wuhan, China) Validated by the company and by users (cited 4 times)<br>CBL (cat#A7881) from ABclonal (Wuhan, China) Validated by the company and in this manuscript (cited 4 times)<br>β-Actin (cat#4970S) from Cell Signaling Technology (MA, USA) Validated by the company and by users (cited 1675 times)<br>GAPDH (cat#2118) from Cell Signaling Technology (MA, USA) Validated by the company and by users (cited 2547 times)<br>osteocalcin (cat#ab13420) from Abcam (Cambridge, UK) Validated by the company and by users (cited 86 times)<br>GFAP (cat#MAB360) from Merck (Darmstadt, Germany) Validated by the company and by users (cited 622 times)<br>A2M (cat#A1573) from ABclonal (Wuhan, China) Validated by the company and in this manuscript (cited 5 times)<br>MAP2 (cat#4542S) from Cell Signaling Technology (MA, USA) Validated by the company and by users (cited 52 times)<br>UCHL1 (Cell Signaling Technology, cat#13179, 1:1000), Validated by the company and by users (cited 8 times)

Eukaryotic cell lines

Policy information about cell lines

Cell line source(s)	MC3T3-E1, HT22, BMSC.
Authentication	The suppliers routinely authenticate the cell lines by short tandem repeat profiling though the cell lines were not authenticated by our laboratory.
Mycoplasma contamination	Mycoplasma test was negative.
Commonly misidentified lines (See ICLAC register)	No commonly misidentified cell lines were used.

Animals and other organisms

Policy information about studies involving animals; ARRIVE guidelines recommended for reporting animal research

Laboratory animals	The male SD rats weighing approximately 250 g (7W) were used.
Wild animals	The study did not involve wild animals.
Field-collected samples	No field-collected samples were used.
Ethics oversight	The experimental protocols were approved by the Animal Care and Use Committee of the Southern Medical University, Guangzhou, China.

Note that full information on the approval of the study protocol must also be provided in the manuscript.

Human research participants

Policy information about studies involving human research participants

Population characteristics	We include data from 24 extensively characterized patients with long-bone fracture (n=12, age range 21-62 years; 7 males, 5 females) and long-bone fracture and concomitant TBI (n=12, age range 25-65 years; 7 males, 5 females). Exclusion criteria included all forms of prior neurological pathology or bone related diseases, immunosuppression, rheumatoid arthritis, and diabetes, as well as steroid or bisphosphonate therapy.
Recruitment	The patients were recruited at the Third Affiliated Hospital of Southern Medical University. Informed consent was obtained from all subjects
Ethics oversight	Ethics approval was granted by the Human Research Ethics Committee of the Third Affiliated Hospital of Southern Medical University.

Note that full information on the approval of the study protocol must also be provided in the manuscript.

Flow Cytometry

Plots

Confirm that:

- [x] The axis labels state the marker and fluorochrome used (e.g. CD4-FITC).
- [x] The axis scales are clearly visible. Include numbers along axes only for bottom left plot of group (a 'group' is an analysis of identical markers).
- [x] All plots are contour plots with outliers or pseudocolor plots.
- [x] A numerical value for number of cells or percentage (with statistics) is provided.

Methodology

Sample preparation	A multi-step centrifugation procedure was used to isolate culture supernatant exosomes, as described previously(Prokopi et al., 2009). Briefly, the collected culture supernatant was pre-purified by centrifugation at 300 × g for 10 min at 4℃ to remove floating cells and debris, and then at 2,000 × g for 20 min to obtain apoptotic body and at 20,000 × g for 30 min at 4℃ to obtain microvesicles. At each step, the supernatant was transferred to new tubes and the pellets immediately resuspended in either phosphate-buffered saline (PBS) or rinsed with PBS. The resulting supernatant was filtered through a 0.22-μm membrane to remove particles larger than 200 nm. sEVs were pelleted via ultracentrifugation at 100,000 × g for 70 min at 4℃.
Instrument	Samples were analyzed using an A-50 Micro-PLUS flow cytometer (Apogee Flow Systems, Hertfordshire, UK).
Software	The data were analyzed using FlowJo version 10.4 software (FlowJo LLC, Oregon, USA).
Cell population abundance	Before sample analysis, calibration of flow cytometer was performed using a reference bead mix (ApogeeMix, Apogee Flow Systems), composed of a mixture of silica nanoparticles with diameters of 110, 180, 240, 300, 590, 880, and 1,300 nm with a refractive index (RI) of 1.42, and 110 and 500 nm green fluorescent (excited by blue laser) polystyrene nanoparticles with an RI of 1.59 (latex) were used.
Gating strategy	110 and 500 nm green fluorescent (excited by blue laser) polystyrene nanoparticles with an RI of 1.59 (latex) were used.

- [x] Tick this box to confirm that a figure exemplifying the gating strategy is provided in the Supplementary Information.
