# Supplementary material for: Damaged brain accelerates bone healing by releasing small extracellular vesicles that target osteoprogenitors
Source: Nat Commun. 2021 Oct 15;12:6043. doi: 10.1038/s41467-021-26302-y (PMC8519911; doi:10.1038/s41467-021-26302-y)
Supplement: Supplementary file 3 — Description of Additional Supplementary Files [file 41467_2021_26302_MOESM3_ESM.pdf]

Title: Supplementary Dataset 1.

Description: Proteins examined in the plasma sEVs of TBI vs. Sham group, which is related to Figure 6.
